# Image fusion guidance for left subclavian artery in situ fenestration during thoracic endovascular repair

**DOI:** 10.1186/s13019-024-02561-w

**Published:** 2024-02-22

**Authors:** Liang Zhao, Jidong Liu, Xiaoshu Cai, Wengang Yang, Ji Wang

**Affiliations:** 1 Department of Radiology, Renji Hospital, School of Medicine, Shanghai Jiaotong University, Shanghai, 200127 P. R. China; 2 Department of Cardiovascular Surgery, Renji Hospital, School of Medicine, Shanghai Jiaotong University, Shanghai, 200127 P. R. China; 3 Advanced Therapies, Siemens Healthineers Ltd, Shanghai, China

## Abstract

**Objectives:**

To evaluate the feasibility and clinical benefit of utilizing image fusion for thoracic endovascular repair (TEVAR) with in situ fenestration (ISF-TEVAR).

**Materials and methods:**

Between January 2020 and December 2020, we prospectively collected 18 consecutive cases with complex thoracic aortic lesions who underwent image fusion guided ISF-TEVAR. As a control group, 18 patients were collected from historical medical records from June 2019 to December 2019. The fusion group involved the use of 3D fusion of CTA and fluoroscopic images for real-time 3D guidance, and the control group involved the use of only regular fluoroscopic images for guidance. The total contrast medium volume, hand-injected contrast medium volume, overall operative time, radiation dose and fluoroscopy time were compared between the two groups. Accuracy was measured based on preoperative CTA and intraoperative digital subtraction angiography.

**Results:**

3D fusion imaging guidance was successfully implemented in all patients in the fusion group. Hand-injected contrast medium volume and overall operative time were significantly lower in the fusion group than in the control group (*p* = .028 and *p* = .011). Compared with the control group, the fusion group showed a significant reduction in time and radiation dose-area product (DAP) for fluoroscopy (*p* = .004 and *p* = .010). No significant differences in total radiation dose (DAP) or total contrast medium volume were observed (*p* = .079 and *p* = .443). Full accuracy was achieved in 8 cases (44%), with a mean deviation of 2.61 mm ± 3.1 (range 0.0-8.4 mm).

**Conclusions:**

3D image fusion for ISF-TEVAR was associated with a significant reduction in hand-injected contrast medium, time and radiation exposure for fluoroscopy and overall operative time. The image fusion guidance showed potential clinical benefits towards improved treatment safety and accuracy for complex thoracic endovascular interventions.

## Introduction

Thoracic endovascular repair (TEVAR) is considered a preferred treatment of choice for thoracic aortic pathologies, including aneurysms, penetrating aortic ulcers, intramural haematomas and dissections [1–3]. Due to the complex anatomy of the aortic arch, dealing with anatomically complex aneurysms or dissections involving one or more neck branches is challenging [4]. In patients who need TEVAR where achievements of a proximal seal necessitates coverage of the left subclavian artery (LSA), revascularization of the LSA is recommended to reduce the risk of perioperative or postoperative stroke, paralysis, and upper extremity ischaemia [5]. Techniques such as chimneys and fenestrated/branched endografts are associated with a high likelihood of endoleaks [6–8] and high costs together with significant manufacturing delays [9, 10], respectively, which has prompted the development of on-site modifications of endografts.

Several studies [11–15] have shown that in situ fenestration (ISF) of the endograft to preserve the LSA during TEVAR has the potential to be a viable alternative to LSA revascularization in recent years, especially in urgent or emergent settings. One of the keys to the success of the TEVAR with in situ fenestration (ISF-TEVAR) procedure is the accurate positioning of the perforation. Image fusion techniques may facilitate this procedure by providing a 3D visualization of the target vessel ostium and landing zone. Previous studies have demonstrated the feasibility of image fusion guidance in standard (thoracic) endovascular repair (EVAR) without LSA coverage [16–19], fenestrated/branched endovascular repair (FEVAR/BEVAR) and chimneys [20–25] or ISF for endovascular repair of complex infrarenal aortic aneurysms (EVARs) [26, 27]. Compared to ISF-EVAR, fusion-guided ISF-TEVAR remains even more challenging due to more pronounced respiratory movements and device-related vessel deformations. However, few studies have reported the efficacy and accuracy of using image fusion in ISF-TEVAR procedures. In the present study, we aimed to evaluate the feasibility and explore the clinical value of image fusion for ISF-TEVAR procedures.

## Materials and methods

### Patient selection

In this single-centre prospective study, we collected 18 consecutive patients with complex aortic disease who underwent image fusion guided ISF-TEVAR between January 2020 and December 2020. The inclusion criteria were patients undergoing LSA ISF-TEVAR due to an insufficient proximal landing zone and requiring intentional coverage of the LSA. One patient with missing radiation report and one patient with concurrent stent placement for renal artery stenosis were excluded. The remaining 16 cases were included as fusion group. In control group, 18 consecutive patients underwent conventional ISF-TEVAR without image fusion were collected from historical medical records between June 2019 and December 2019. All cases included were performed by the same operation team and one-year follow-up data was also collected for each patient. This study was approved by the ethics committee from the institutional review board of Renji Hospital (No. 102 K), and informed consent was obtained from each prospectively patient.

### Preprocedural CTA imaging

**Fig. 1:**
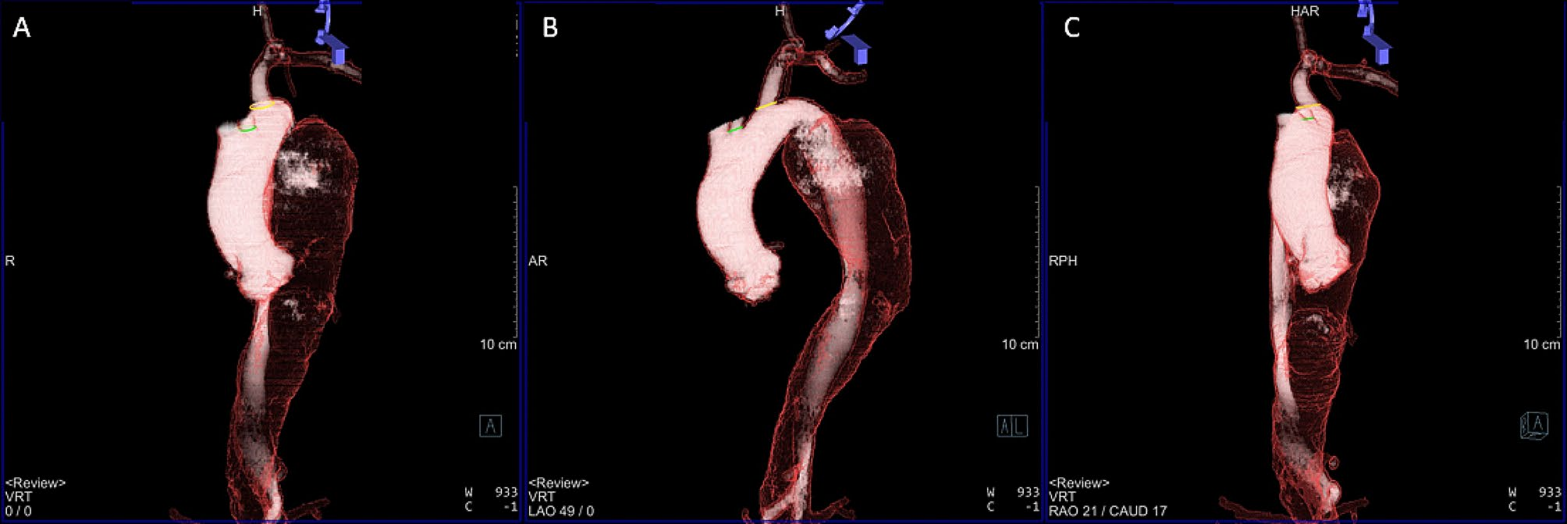
3D reconstruction of CTA with landmarks of ostia in the (**A**) anteroposterior view and (**B**) arch view, showing the optimum working angle with full visualization of LSA and LCCA, and in the (**C**) en face view to visualize the proximal shape of LSA. The white solid area indicates the true lumen, and the transparent area indicates the false lumen

All patients underwent preoperative contrast-enhanced multi-slice CT scans (Aquilion One 320, Toshiba Medical Systems, Nasu, Japan) with a slice thickness of 0.5 mm for measurements and with a position of bilateral upper extremity elevation. The CT scan was usually being planned less than a week before the procedure. The 3D reconstruction and segmentation of the thoracoabdominal aortic system and its major branches were performed in advance on a CT workstation (Fig. 1). The reconstruction slice thickness was 0.5 mm. Prior to the intervention, the segmented 3D images were loaded to a dedicated workstation (syngo X Workplace, Siemens Healthineers), which was connected to the operating C-arm system. Landmarks such as target vessel ostia, planned proximal landing zones and other operative landmarks of interest were added as 3D images to the volume-rendering techniques (VRTs) (Fig. 1). Additionally, based on these landmarks, the operator could evaluate the optimal parallax correction angle with full visualization of the LSA and the left common carotid artery (LCCA), which could be automatically transferred to the C-arm system.

### CBCT imaging acquisition

All procedures were performed under a flat-panel detector C-arm angiography system (Artis Zeego, Siemens Healthcare, Germany). After induction of anaesthesia and patient preparation in the hybrid operating room, a 10-second unenhanced cone beam computed tomography (CBCT) was performed to obtain imaging of the aortic arch. The protocol captured 296 frames in 10 s during a 200° C-arm rotation, with the detector in landscape orientation.

### Image registration

**Fig. 2:**
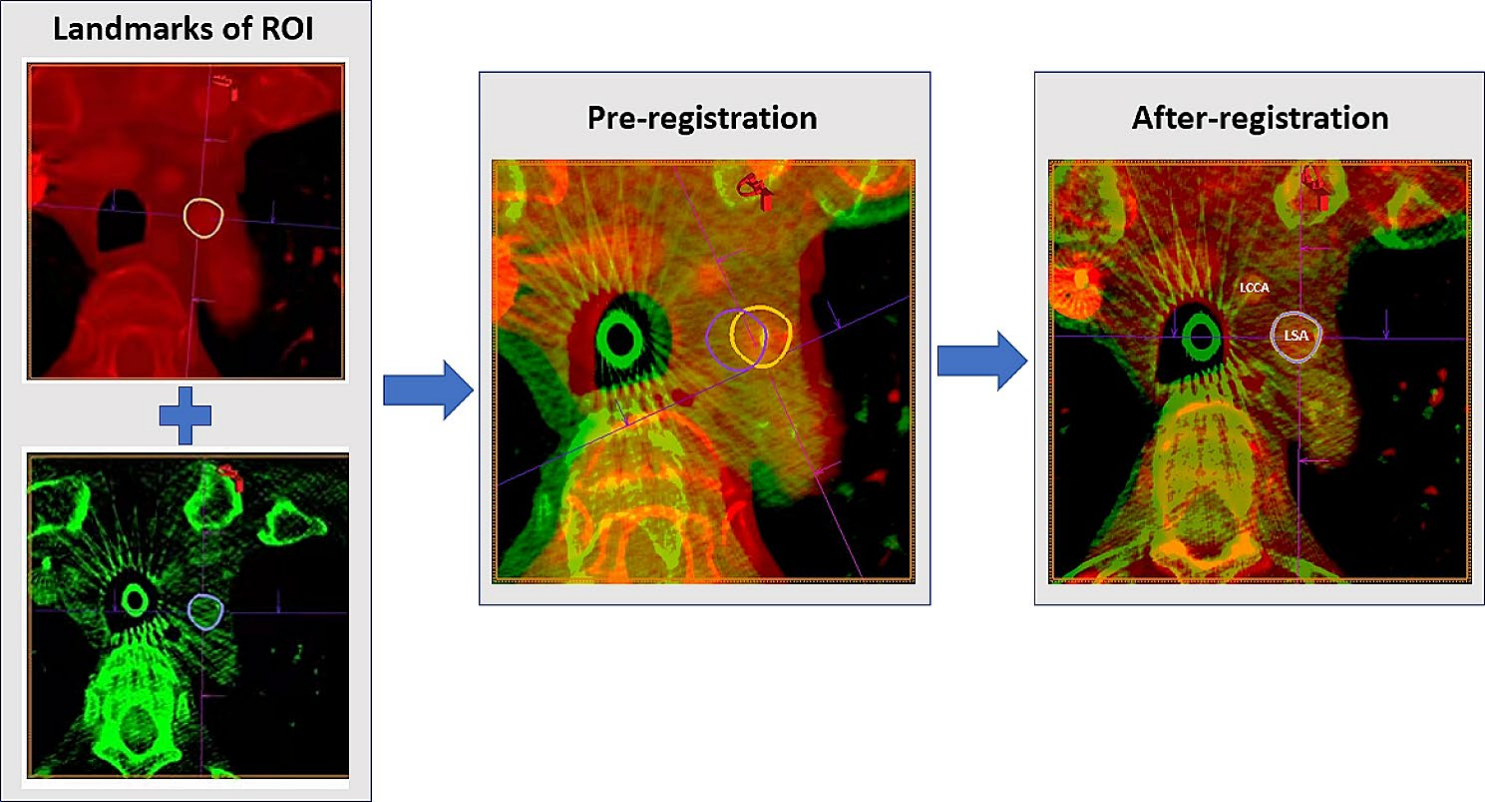
Image registration of preoperational three-dimensional CTA (red modality, upper left) and intraoperative CBCT (green modality, bottom left). First, the LSA ostium (the ROI in this study) was marked in both CTA (yellow circle) and CBCT (purple circle) modalities. Second, automatic registration, which was based on bony landmarks by default, could be performed to ensure the two modalities are in the same field of view. Thirdly, landmarks of the LSA were aligned from coronal view and checked from other planers to complete the registration

Image fusion was applied using an advanced application (*syngo* InSpace 3D/3D fusion; Siemens Healthineers) to coregister CBCT images with preoperative CTA images (Fig. 2). First, the region of interest (ROI), which was the LSA ostium in this study, was marked in CTA and CBCT modalities, respectively. In a second step, the two modalities could be automatically registered based on bony structures to ensure the two modalities are in the same field of view. Subsequently, in a third step, we proposed a novel soft tissue-based registration method that aligns CTA and CBCT images based on contours of soft tissue or calcified tissue landmarks within the region of interest rather than the simply used bony landmarks. As a closer alignment, two modalities could be finely tuned and aligned according to the soft tissue contour of aorta arch and landmarks of the LSA on orthogonal multiplanar reconstruction (MPR) images in axial, sagittal and coronal orientations. If calcifications presented, calcification landmarks could also be used to facilitate the registration. Fusion processes were performed during patient preparation for surgery after general anaesthesia. CTA and CBCT images were semiautomatically registered in less than 2 min.

### Intraoperative overlay

**Fig. 3:**
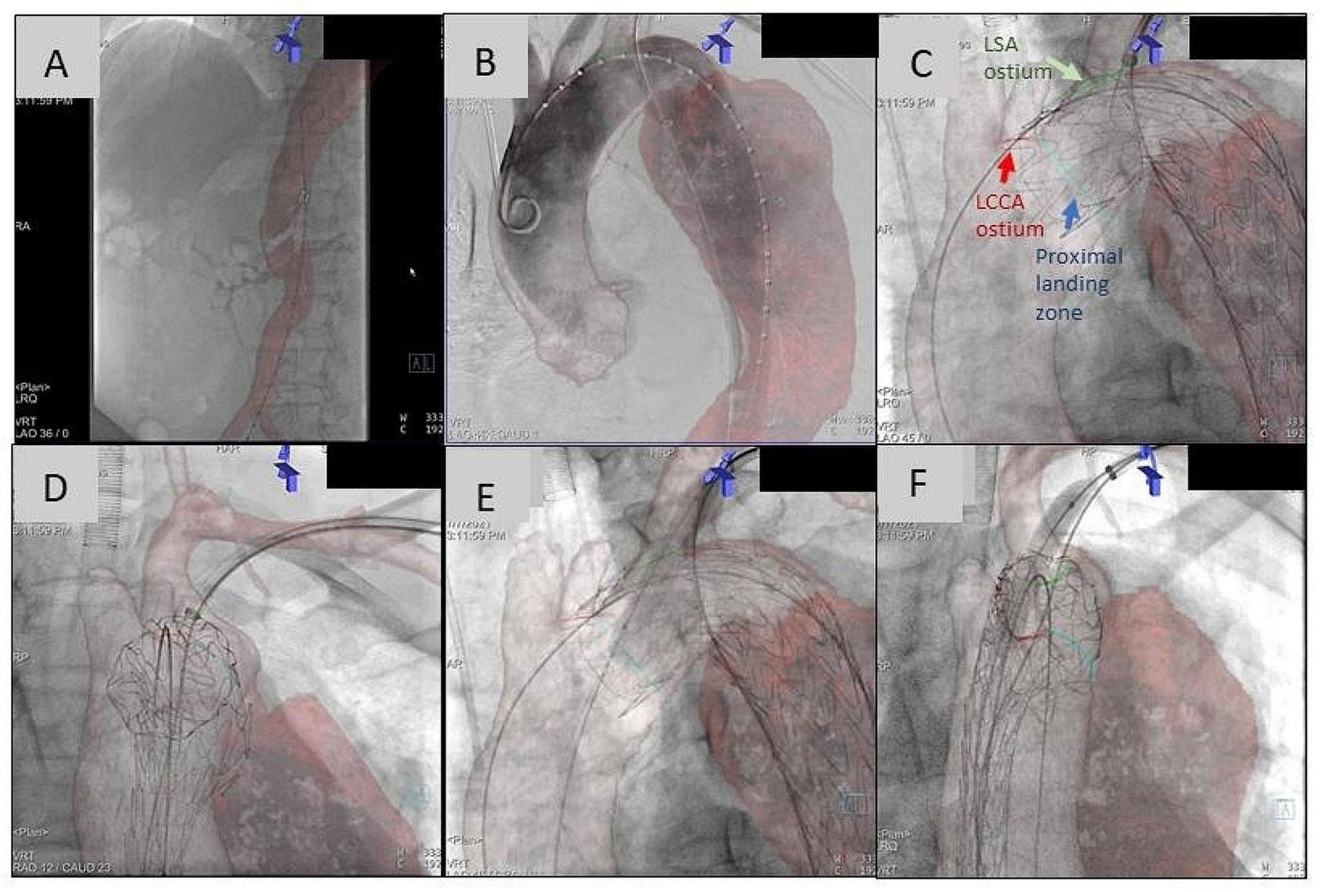
Representative case showing intraprocedural guidance under image fusion. (**A**) The device was introduced along the true lumen of the abdominal aorta, clearly displayed with fused CTA imaging. (**B**) The device reached the aortic arch with contrast injection, which confirmed the accuracy of the fused image. (**C**) Deployment of graft stent and perforation under image fusion guidance at an optimal working angle (arch view). Image fusion showing the centre of the LSA ostium to facilitate the accurate positioning of perforation. (**D**) Deployment of graft stent and perforation under image fusion guidance (en face view). Real-time three-dimensional fusion guidance reduced repeated angiographies between two common working angles: (**E**) predilation of balloon under image fusion guidance (arch view); (**F**) predilation of balloon under image fusion guidance (en face view). LSA: left subclavian artery; LCCA: left common carotid artery

3D anatomical projections and target vessel landmarks premarked on CTA images could be superimposed on 2D fluoroscopic images (Fig. 3). These landmarks were synchronized with the 3D model, providing real-time updates of the 3D visualization as the C-arm angle and table motion changed. Due to aortic arch branch puncture during ISF, the proximal LSA anatomy may be deformed by the stiff devices. Final alignment with intraoperative arch-view angiography, which was used to evaluate the preoperative aortic blood flow, was used to adjust overlay images and confirm the accuracy of fusion. After confirmation, the C-arm angle was directly adjusted to the precalculated optimal parallax correction position, without repeating angiographic scans.

### Intervention under fusion guidance

All patients were under general anaesthesia. Percutaneous femoral access was obtained, followed by introduction of a pigtail catheter along the true lumen of the aorta under guidance of image fusion (Fig. 3A). The pigtail catheter was advanced all the way to the ascending aorta for further angiography and stent graft placement. With a surgical incision, the left brachial artery (LBA) was exposed directly. A long sheath (COOK MEDICAL, Flexor Check-Flo Introducer, 9 F, 55 cm in length/ GORE, DrySeal Flex Introducer Sheath, 10 F, 65 cm in length) was inserted into the LBA, and the tip was kept at the LSA ostium. A 4Fr MPA catheter (Cordis, 125 cm in length) with Amplatz Super Stiff guidewire (Boston Scientific-TIP, 0.035/0.018 in diameter, 260 cm in length) was advanced from the LBA long sheath to form an “anterior junction” (Fig. 3B) to the pigtail catheter. The 4Fr MPA catheter was further safely extended to the distal true lumen of the descending aorta (usually below the renal artery level) based on image fusion guidance (Fig. 3B). The Super Stiff guidewire with the 4Fr MPA catheter formed a “traction system” for the long sheath. Under an optimal working angle provided by fusion image, the aortic stent graft (Medtronic, VALIANT THORACIC Stent Graft with the Captivia Delivery System; Lifetech, Ankura; GORE) was inserted through femoral access and deployed with the guidance of the suggested proximal landing zone marker (Fig. 3C). Afterwards, the puncture needle (OLYMPUS MEDICAL SYSTEM CORP, Model NA-201SX-402, 21G/ OLYMPUS MEDICAL SYSTEM CORP, Model NA-220 H-8019, 19G) was introduced through the long sheath down to the ostium of the LSA along with the “traction system”. The puncture angle and needle position were adjusted and confirmed guided by fusion image to ensure that the perforation was in the centre of the LSA ostium as much as possible (Fig. 3C-D) with a reduced contrast medium used.

After successful fenestration, a 5 mm percutaneous transluminal coronary angioplasty (PTCA) balloon (Boston Scientific, MUSTANG) was used to enlarge the puncture site (Fig. 3E-F). Subsequently, an 8 mm balloon was used for further dilation. Along with the stiff guidewire, a covered self-expanding stent (BARD, FLUENCY plus Vascular Stent Graft, 40 mm in length) was applied through the long sheath and deployed at an optimal position. A final 2D angiogram was acquired to confirm the successful deployment of the main graft and LSA revascularization.

### Outcome parameters

**Fig. 4:**
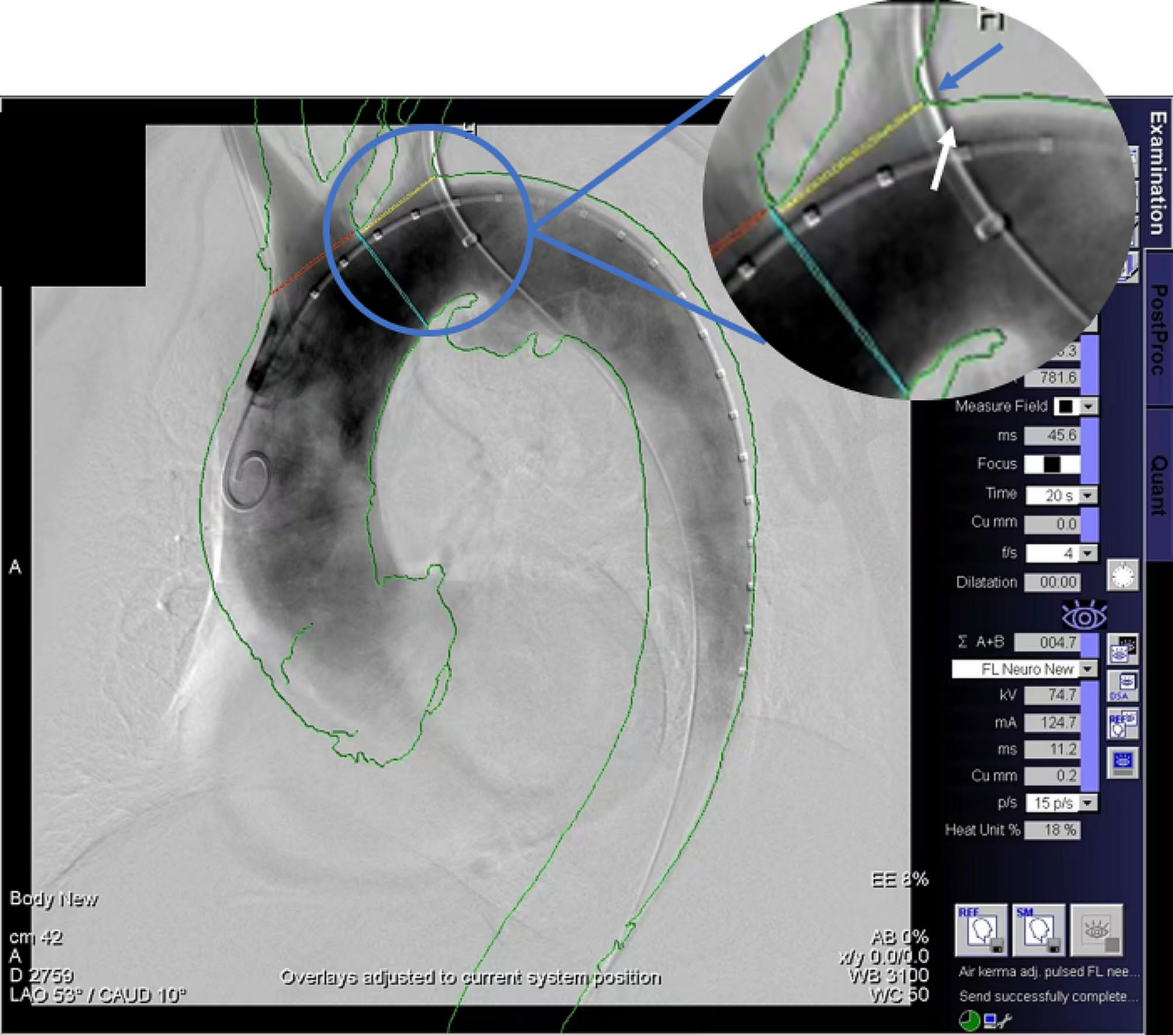
Fusion landmarks are outlines: contrasted lumen (green), LSA ostium (yellow circle), LCCA ostium (red circle) and proximal landing zone (blue-green circle). Blue arrow: suggested intersection angle of LSA ostium and aortic based on fusion imaging; White arrow: real intersection angle of LSA ostium and aortic arch based on DSA. The deviation of fusion was defined as distance between suggested and real inflection point of intersection angle between LSA ostium and aortic arch. LSA, left subclavian artery; LCCA: left common carotid artery

**Fig. 5:**
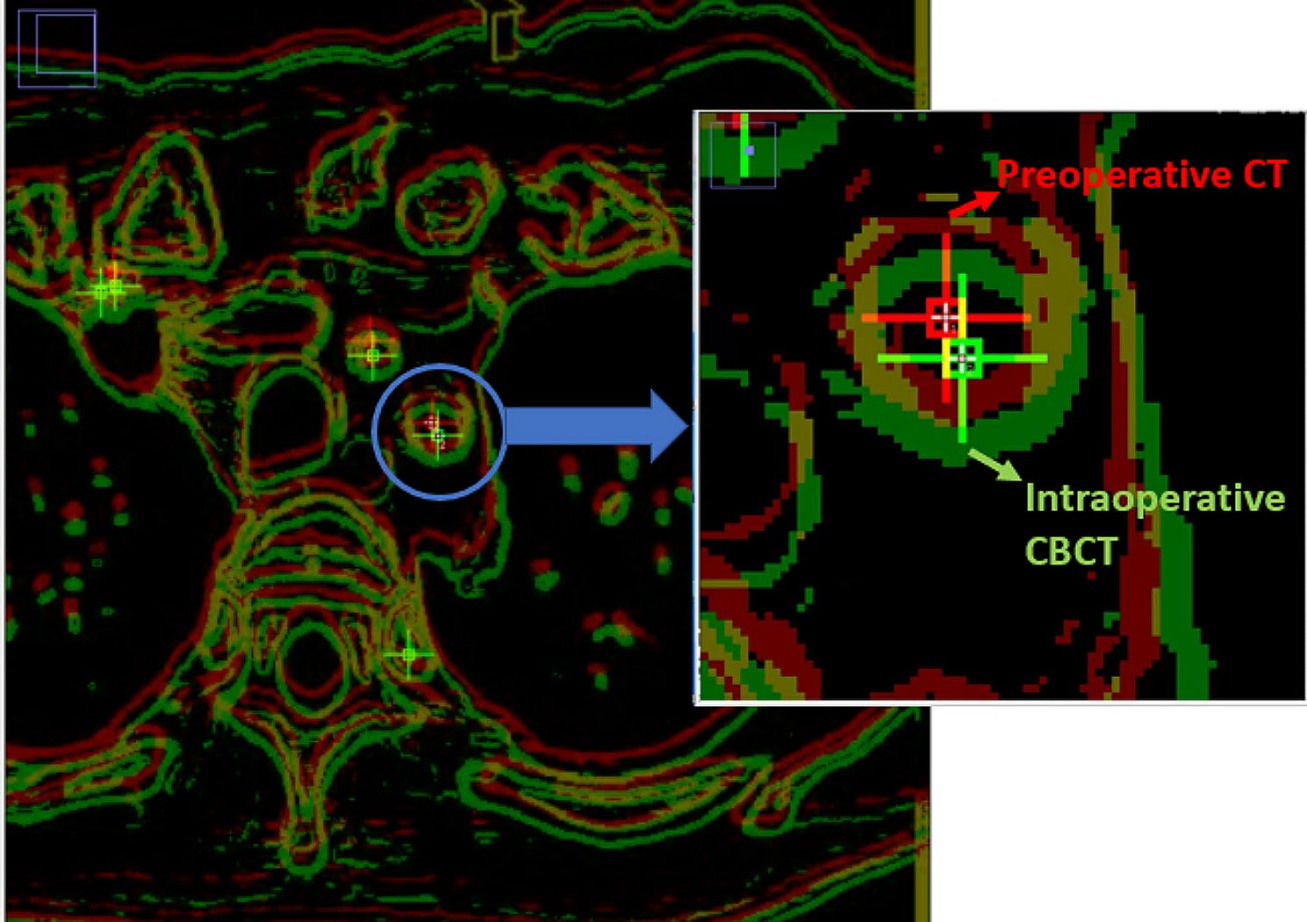
The distance between the center of LSA ostium between preoperative CTA (red cross cursor) and intraoperative CBCT (green cross cursor) imaging was measured using ImageJ. The deviation derived from automatic bony-based registration and soft tissue-based registration method could be computed, which indicated the improvement of mismatch in terms of the ROI (LSA ostium) in using novel soft-tissue method

The main parameters we measured between the two groups were the radiation dose-area product (DAP, in Gy · cm^2^) in fluoroscopy and, in total, the volume of contrast medium, overall operative time and fluoroscopy time. Image accuracy was recorded as the difference between CTA images and angiographic contours during intraoperative overlays. The deviation was measured as the distance between the inflection point of intersection angle between the LSA ostium and aortic arch on fused CTA image and intraoperative angiography, according to the measurement method described in the report by Schulz et al. [16] (Fig. 4). By our definition, full accuracy was achieved when the inflection point of intersection angle of the fused CTA image exactly overlapped that of intraoperative DSA. To better understand the improvement of soft tissue-based method compared to traditional bony-based method, we measured the mismatch reduced of the center of LSA ostium after using soft-tissue based method. We computed the difference of LSA center deviation in preoperative CTA and intraoperative CBCT imaging between these two methods (Fig. 5). Image contour extraction, synthesis of reconstruction and distance measurement were performed using ImageJ (version 1.48v, National Institutes of Health).

### Statistical analysis

Continuous variables are expressed as means ± standard deviations for variables normally distributed and as median with interquartile range (IQR) for skewed variables. Categorical variables are presented as absolute values and percentages. Statistical analysis was performed with the Statistical Product and Service Solutions (SPSS) statistical software program (version 22.0, IBM, Chicago). We assessed the differences between two groups using the χ^**2**^ test or Fisher’s exact test for categorical variables. For continuous variables, a 2-sided Student’s t test for normally distributed variables or the Mann–Whitney U test for nonparametric variables was used. Statistical significance was set to 5%.

## Results

### Patient characteristics

The fusion group consisted of 14 males and 2 females, with a mean age of 61 ± 12 years. The control group consisted of 16 males and 2 females, with a mean age of 59 ± 12 years. Comparable demographic data were observed in the two groups. The detailed patient characteristics are shown in Table 1.

**Table 1:** Patient characteristics

Characteristics	*Fusion group (n = 16)*	*Control group (n = 18)*	*P* value
Age (years)	61 ± 12	59 ± 12	0.910
Female: n (%)	2 (12.5%)	2 (11.1%)	0.900
BMI (kg/m^2^)	26.6 ± 3.5	26.2 ± 5.9	0.877
Pathology: n (%)			0.144
IMH	3 (18.8%)	1 (5.6%)	
AD	7 (43.8%)	3 (16.7%)	
PAU	7 (43.8%)	15 (83.3%)	
TAA	1 (6.3%)	1 (5.6%)	
Aortic Arch Type [28]: n (%)			0.341
Type I	2 (12.5%)	5 (27.8%)	
Type II	11 (68.8%)	8 (44.4%)	
Type III	3 (18.7%)	5 (27.8%)	

*IMH: intramural haemorrhage and haematoma; AD: aortic dissection; PAU: penetrating aortic ulcer; TAA: thoracic aortic aneurysm

Values are expressed as mean ± SD or number (%)

### Procedure outcomes

Successful fenestrations under fusion guidance were achieved in all 16 patients in the fusion group. The overall operative time was significantly lower in the fusion group than in the control group (81.1 min ± 20.7 vs. 91.1 min ± 21.6, *p* = .011). The mean volume of hand-injected contrast medium was 15.1 mL ± 5.7, which was also significantly lower than that of the control group (23.7 mL ± 10.8, *p* = .028). In addition, the fusion group showed a significant reduction in fluoroscopy time and radiation dose for fluoroscopy (DAP-fluoro) compared with those of the control group (21.7 min ± 4.4 vs. 33.7 min ± 14.0, *p* = .010 and 184.7 (119.1–271.5) Gy · cm^2^ vs. 257.9 (181.6–342.5) Gy · cm^2^, *p* = .004). No significant differences in total radiation dose (DAP-total) or total contrast medium volume were observed (473.8 (345.3–666.7) Gy · cm^2^ vs. 510.1 (416.0–727.5) Gy · cm^2^, *p* = .079 and 163.9 mL ± 17.3 vs. 170.4 mL ± 16.4, *p* = .443). Additional results are described and summarized in Table 2.

**Table 2:** Procedural outcome results

Outcomes	Fusion group	Control group	*P* value
Operative time (min)	81.1 ± 20.7	91.1 ± 21.6	0.011
Fluoroscopy time (min)	21.7 ± 4.4	33.7 ± 14.0	0.010
DAP (Gy · cm^2^)-fluoro	184.7 (119.1–271.5)	257.9 (181.6–342.5)	0.004
DAP (Gy · cm^2^)-total	473.8 (345.3–666.7)	510.1 (416.0–727.5)	0.079
Total contrast medium volume (mL)	163.9 ± 17.3	170.4 ± 16.4	0.443
Hand-injected contrast medium volume (mL)	15.1 ± 5.7	23.7 ± 10.8	0.028

Values are expressed as median with IQR or mean ± SD

### Image fusion accuracy

With respect to fusion accuracy, full accuracy, indicating a complete alignment between the fused CTA imaging and the intraoperative DSA run, was achieved in 8 cases (44%). The median deviation was 1.45 mm (range 0.0-8.4 mm), with a mean deviation of 2.61 mm. By comparing the difference of LSA center deviation in preoperative CTA and intraoperative CBCT imaging between soft tissue-based method and bony-based method, the median deviation was 19.7 mm (range 13.0–28.6), which indicated the mismatch corrected by soft tissue-based method.

### Follow-up results

All patients received regular CTA follow-up at 3, 6, and 12 months after discharge. Both groups had already produced follow-up images and clinical data within one year.

All patients survived. No major neurologic or left upper extremity ischaemia events were observed. All branch stents and aortic grafts were unobstructed, and no migration or destruction was found. The mean inner diameter of the LBA branch stent at the junction site with the aortic graft was 5.5 mm (3.8–8.7 mm). Endoleaks occurred in three cases at the one-year follow-up. There was one case of a type 2 endoleak in both the fusion group and the control group, and the aorta was not dilated. One case in the control group had a type 1b endoleak.

## Discussion

This study showed that the use of fusion image guidance is associated with significant reductions in overall operative time, contrast medium volume, fluoroscopy time, and radiation dose for fluoroscopy in ISF-TEVAR. In addition, it demonstrated that perfect fusion accuracy was achieved in 8 out of 15 cases (44%), with a satisfactory deviation in most patients. The key advantage of fusion guidance is to provide a safe and accurate perforation localization to ensure successful fenestration. To the best of our knowledge, this is the first comparative study in which this image fusion technique is used for ISF-TEVAR.

Previous studies have demonstrated the feasibility and accuracy of fusion imaging for standard TEVAR procedures without LSA revascularization [17, 18, 26]. Performing a subgroup analysis, Dias et al. first showed in a comparative study [29] that image fusion guidance could significantly reduce the total radiation dose in standard TEVAR procedures, even in only ten patient samples. Subsequently, Hiraoka et al. further demonstrated that significant reductions in exposure and contrast medium could be achieved by the use of image fusion in standard TEVAR procedures involving larger populations [30]. For complex aortic disease, image fusion guidance has been primarily described in endovascular repair procedures. A meta-analysis suggested that image fusion could significantly lower the contrast volume, fluoroscopy time and operative time in complex (fenestrated/branched) EVAR [25]. Regarding ISF, Leger et al. first reported a cohort study demonstrating the feasibility and benefit of ISF for complex EVAR guided by 3D image fusion. Image fusion guidance has rarely been used for ISF-TEVAR requiring LSA revascularization. By providing real-time visualization of 3D vascular structure, the current comparative study indicated that exposure and operative time could also be reduced with ISF-TEVAR.

Problems associated with inaccuracies have been thoroughly discussed in Schulz’s [16] and Sailer’s [23] studies. The reasons could be grouped into three main categories: different patient positions between CTA and intraoperative CBCT, patient movement, and straightening and deformation of vessels. In response to the first problem, CTA acquisition needs to be performed with normal breathing followed by breath holding, which is similar to the case of general anaesthesia with low tidal volume ventilation. Additionally, patient repositioning was avoided after CBCT acquisition to eliminate inaccuracies in patient movement. The most challenging inaccuracies originate from deformations caused by the insertion of stiff devices, including guidewires and introducers. In our centre, we proposed a novel registration method that aligns CTA and CBCT images based on contours of soft tissue or calcified tissue landmarks within the region of interest rather than the traditionally used bony landmarks. A closer alignment to the region of interest could reduce the mismatch caused by respiratory diseases, especially for cases such as TEVAR procedures. In addition, multiplanar registration brought to a more accurate three-dimensional alignment instead of two-dimensional manual adjustment after overlaying. Therefore, this registration method greatly improved the accuracy of fusion. Compared to the accuracy reported in Schulz’s [16] study for the standard TEVAR procedures without LSA revascularization, the median deviation in our study on ISF-TEVAR was eight times more accurate (1.45 mm, range 0.0-8.4 vs. 11.7 mm, range 0.0-37.2). The main reason could be that the 3D-3D registration method used in this study was based on soft tissue instead of bony structures.

Fusion-guided ISF-TEVAR may provide several benefits for operators. (i) By reconstructing and overlaying the 3D vascular anatomy of the region of interest, image fusion guidance could help operators increase the visibility of important structures. Even though most of vascular anatomy might be greatly deformed by insertion of stiff device, the region of interest like targeted ostium could still be helpful after readjustment and reconfirmation. Instead of the guidance of traditional 2D angiograms, which require multiple rounds of confirmation from different angles, the guidewires and devices can be guided in a more intuitive way through 3D visualization. Accordingly, fluoroscopy time could be significantly reduced by accelerating the process of fenestration. (ii) The overlaid vascular anatomy provided visualization of the true and false lumen, which could facilitate the guidewire keep introducing along the true lumen and reduce the potential risk of introducing into the false lumen. This way, contrast medium for confirming the position of devices could also be reduced significantly. (iii) Image fusion could assist operators in obtaining the optimal working angle under fluoroscopy and angiography in a simpler manner and in less time. Determining the optimal working angle is key to precise stent deployment and perforation. An optimal working angle satisfies the following criteria: (1) the LSA and LCCA can be fully visualized, which helps the stent to be deployed accurately, and (2) the plane of the LSA ostium is on top of the aortic arch such that the perforation site is displayed in a more intuitive way. Image fusion with a three-dimensional aortic arch display may provide a simpler way to find the optimal working angle, thereby reducing the unnecessary radiation dose for both patients and operators. iv) The image fusion technique is especially beneficial to novice operators when intuitive visualization can advance their understanding of DSA imaging with anatomy, potentially hastening the learning curve of the procedure.

However, we should also acknowledge some limitations. First, this is a single-centre study with a small sample size. It is possible that with increased experience of this type of procedure, the operative time and radiation dose might be also improved accordingly, and the significance of the image fusion need to be further investigated. Therefore, a larger, multicentre study will be needed in the future. Second, when the stiff needle system is inserted, the target artery like LSA would encounter inevitable deformation. Thus, before image-guided puncture, it is essential to reconfirm the position of the puncture site and the left vertebral artery bifurcation when puncturing the graft and implanting the device.

## Conclusion

In conclusion, this comparative study confirmed that the use of image fusion could significantly reduce the hand-injected contrast volume, fluoroscopy time, radiation exposure and operative time for ISF-TEVAR. Furthermore, soft tissue-based registration was shown to be potentially advantageous in clinical cases and allows for better adjustment. The image fusion guidance showed potential clinical benefits towards improved treatment safety and accuracy for complex thoracic endovascular interventions.

## Data Availability

The datasets used and/or analysed during the current study available from the corresponding author on reasonable request.
